# Bimodal modulation of L1 interneuron activity in anterior cingulate cortex during fear conditioning

**DOI:** 10.3389/fncir.2023.1138358

**Published:** 2023-06-02

**Authors:** Giuliana Fossati, Daniel Kiss-Bodolay, Julien Prados, Ronan Chéreau, Elodie Husi, Christelle Cadilhac, Lucia Gomez, Bianca A. Silva, Alexandre Dayer, Anthony Holtmaat

**Affiliations:** ^1^Department of Basic Neurosciences, and Neurocenter, Faculty of Medicine, University of Geneva, Geneva, Switzerland; ^2^Neuro Center, IRCCS Humanitas Research Hospital, Milan, Italy; ^3^Department of Neurosurgery, Geneva University Hospitals, Geneva, Switzerland; ^4^Lemanic Neuroscience Doctoral School, University of Geneva, Geneva, Switzerland; ^5^Department of Psychiatry, University of Geneva, Geneva, Switzerland; ^6^National Research Council of Italy, Institute of Neuroscience, Milan, Italy

**Keywords:** anterior cingulate cortex, serotonin receptor 3a, neurogliaform cells, fear learning, 2-photon laser scanning microscopy, microprism

## Abstract

The anterior cingulate cortex (ACC) plays a crucial role in encoding, consolidating and retrieving memories related to emotionally salient experiences, such as aversive and rewarding events. Various studies have highlighted its importance for fear memory processing, but its circuit mechanisms are still poorly understood. Cortical layer 1 (L1) of the ACC might be a particularly important site of signal integration, since it is a major entry point for long-range inputs, which is tightly controlled by local inhibition. Many L1 interneurons express the ionotropic serotonin receptor 3a (5HT3aR), which has been implicated in post-traumatic stress disorder and in models of anxiety. Hence, unraveling the response dynamics of L1 interneurons and subtypes thereof during fear memory processing may provide important insights into the microcircuit organization regulating this process. Here, using 2-photon laser scanning microscopy of genetically encoded calcium indicators through microprisms in awake mice, we longitudinally monitored over days the activity of L1 interneurons in the ACC in a tone-cued fear conditioning paradigm. We observed that tones elicited responses in a substantial fraction of the imaged neurons, which were significantly modulated in a bidirectional manner after the tone was associated to an aversive stimulus. A subpopulation of these neurons, the neurogliaform cells (NGCs), displayed a net increase in tone-evoked responses following fear conditioning. Together, these results suggest that different subpopulations of L1 interneurons may exert distinct functions in the ACC circuitry regulating fear learning and memory.

## Introduction

Fear and anxiety-related disorders impose a major burden on people and society. Despite the high prevalence of these disorders, their mechanistic underpinnings remain largely unknown. Defensive responses upon perception of fearful stimuli are in large part innate, but they can also be learned through the association of threats and harmful events with concomitantly presented stimuli (Gross and Canteras, 2012; Silva et al., 2016; LeDoux and Daw, 2018).

The neuronal circuits underlying fear and fear learning have been studied in animals and humans (Tovote et al., 2015), mostly using Pavlovian fear conditioning (FC) paradigms. In these paradigms, a mild electric foot shock is typically used as an unconditioned stimulus (US) that elicits an innate fear response. Repeated pairing of the US with a neutral stimulus (conditioned stimulus–CS) leads to a conditioned response (CR) upon subsequent presentation of the CS alone, which in rodents presents itself as freezing behavior. Such studies have revealed major roles for the amygdala, the hippocampus, and various cortical structures in fear learning and memory (Herry and Johansen, 2014; Tovote et al., 2015; Greco and Liberzon, 2016). Of these, high order prefrontal cortical areas were found to play important roles in assessing the severity and controllability of stressors or threats and to bidirectionally modulate fear expression, fear memory encoding and extinction (Amat et al., 2005; Giustino and Maren, 2015).

The medial prefrontal cortex (mPFC) comprises various executive centers implicated in the control of cognitive, emotional and social behaviors (Yizhar et al., 2011; Coley et al., 2021; Howland et al., 2022). Specifically, all its three subdivisions, the infralimbic (IL), prelimbic (PL) and anterior cingulate cortices (ACC), represent crucial nodes in the cortico-hippocampal-amygdala circuit that regulate fear learning and have been selectively implicated in the acquisition, retrieval and extinction of fear memories (Dejean et al., 2015). While the functional circuit organization of the PL and IL subdivisions in relation to fear learning has been investigated in detail, including layer and cell type specific interrogation of function (Courtin et al., 2014; Dejean et al., 2015), the contribution of the ACC to fear learning has been dissected with far less detail. Specifically, the ACC shows a global activity increase upon fear learning in both rodents and humans (Etkin et al., 2011; Steenland et al., 2012; Greco and Liberzon, 2016; Roy et al., 2022). Direct stimulation of the ACC in mice produces long-term fear memories, while its inhibition impairs them (Tang et al., 2005; Einarsson and Nader, 2012; de Lima et al., 2022). However, due to its location deep in the medial bank of the cortex, imaging and electrophysiology studies have mostly been performed at a relatively low resolution and without clear identification of the cell types that displayed activity upon fear conditioning. Therefore, the specific contribution of the different neuronal cell types to the global fear memory-related increase of ACC activity have not yet been disentangled. In particular, the role of inhibitory interneuron types remains elusive, yet they are critical for cortical processing. For example, inhibitory microcircuits in a number of limbic areas have been identified as key regulators of the amount of excitation that a particular stimulus elicits and are essential modulators of the CS-US association coding (Ehrlich et al., 2009; Donato et al., 2013; Courtin et al., 2014; Wolff et al., 2014; Tovote et al., 2015). Therefore, characterizing the evolution of interneuron activity is important for understanding how the ACC processes and encodes information over the course of fear learning.

Interneurons in cortical layer 1 (L1) play an important role in balancing the long-range and local excitation (Tremblay et al., 2016; Ibrahim et al., 2021) and have been shown to regulate fear learning processes (Letzkus et al., 2011, 2015). This suggests that L1 interneurons in the ACC could also play a fundamental role in fear learning and memory. In addition, a subpopulation of L1 interneurons has been shown to express the ionotropic serotonergic receptor 3A (5HT3aR) (Zhou and Hablitz, 1999; Ferezou et al., 2002; Lee et al., 2010; Vucurovic et al., 2010; the Allen Cell Type database: https://portal.brain-map.org/atlases-and-data/rnaseq), a gene which has been linked to fear-related pathologies by genetic and epigenetic studies (Kelmendi et al., 2016; Perroud et al., 2016; Schechter et al., 2017). However, unlike in sensory cortices (Letzkus et al., 2011, 2015) there is a paucity of information about the activity of L1 interneurons in the prefrontal cortex in general and during fear learning in particular. L1 cortical interneurons can be divided into two anatomically and functionally distinct classes: single bouquet cells and neurogliaform cells (NGCs) (Lee et al., 2015). The former display distinct morphological and electrophysiological properties, are characterized by a simple axonal arbor, and preferentially target deeper layer interneurons (Schuman et al., 2019); the latter are characterized by axonal arbors restricted to L1 and contact both interneurons and principal neurons distal dendrites (Letzkus et al., 2011; Jiang et al., 2013; Abs et al., 2018; Niquille et al., 2018; Schuman et al., 2019). NGCs are part of a population of interneurons that have been identified in the auditory cortex as regulators of plasticity in fear conditioning (Abs et al., 2018). However, their activity during fear learning has never been specifically investigated in the ACC.

Here, we utilized transgenic mice and AAV vectors to drive the expression of genetically encoded calcium sensors in the 5HT3aR and NGCs subpopulations of L1 interneurons, and applied 2-photon laser scanning microscopy (2PLSM) through microprism implants to longitudinally image these neurons in the ACC before and after cued FC (Andermann et al., 2013; Low et al., 2014; Pattwell et al., 2016). We show that FC changes their response dynamics. Within both populations, we identified distinct neuronal assemblies that either increase (positively modulated) or decrease (negatively modulated) their responses to the CS following FC and found more positively modulated neurons among the NGC population. These results suggest that fear learning is associated with a net increase in inhibitory activity of a cell type that targets pyramidal cell apical dendrites, potentially impacting the integration of long-range inputs that project to L1 of the ACC.

## Materials and methods

### Animals and viral vectors

Animals were group housed at the University of Geneva’s animal husbandry facility on a 12-h light cycle (lights on at 8:00 a.m.). All procedures were carried out in accordance with protocols approved by the ethics committee of the University of Geneva and the authorities of the Canton of Geneva (license numbers GE/28/14, GE/61/17).

For all the experiments we used 12 weeks-old male mice. To target 5HT3aR-expressing interneurons we used the Tg(Htr3a-cre)NO152Gsat line produced by the Gene Expression Nervous System Atlas [GENSAT] project at the Rockefeller University (New York, NY) (MGI: 5435492; J:100256).^1^ To target NGCs, we used an in-house bred NGC-Flippase line. This line was created by crossing the Tg(Hmx3-icre)1Kess line (MGI: 5566775; MGI:5566775)^2^ (Niquille et al., 2018), with the Cre-conditional Rosa-26-CAG-LSL-Flp line [B6;129S4-Gt(ROSA)26Sortm5(CAG-flpo)Zjh/J].^3^ This strategy allowed robust and local viral vector-mediated expression of a genetically encoded calcium sensor, which was preferable to direct crossing of the Hmx3-Cre line with a Cre-dependent reporter line. For the L1 5HT3aR cell targeting experiments, AAV9-hSyn-DIO-GcaMP6s was injected (Penn Vector Core, PA, USA; 90 nl per injection). For the L1 NGC cell targeting experiments, AAV1/2-hSyn.fDIO.GCaMP6s was injected (EMBL viral core facility, Rome, Italy; 90 nl per injection).

### Surgery for microprism implantation

All surgeries were performed in a dedicated separate surgery room equipped with an intermediary animal housing station and heating pads. Before the surgery, all micro instruments and the surgical bench were cleaned with 70% ethanol (Sigma). Anesthesia induction was done with a mix of O2 and 4% isoflurane at 0.4 L/min (Baxter) followed by an intraperitoneal injection of MMF solution, consisting of 0.2 mg/kg medetomidine (Dormitory, Orion Pharma, Milan, Italy), 5 mg/kg midazolam (Dormicum, Roche, Basel, Switzerland), and 0.05 mg/kg fentanyl (Fentanyl, Sintetica, Münster, Germany) diluted in sterile 0.9% NaCl producing deep anesthesia and analgesia for a minimum of 2 h. After stable deep anesthesia was confirmed by using toe pinching, a 2 μg/g IP injection of dexamethasone was given to reduce brain swelling and inflammation (Mephameson, Mepha Pharma, Aesch, Switzerland). Animals were then placed on a heating pad (MIO Star thermocare 100) set at 37°C and head fixed in stereotaxic frame (Stoelting) equipped with one-axis oil hydraulic micromanipulator (MO-10, Narishige, London, United Kingdom). Sterile lubricant eye ointment was applied (Lacryvisc, Alcon, Geneva, Switzerland). Betadine (Mundipharma, Cambridge, United Kingdom) skin surface disinfection followed by local anesthesia using subcutaneous scalp injection of Lidocaine 1% (Streuli, Uznach, Switzerland) was performed before skin incisions were made. After termination of the surgery, a wake solution composed of Atipamezole 2.5 mg/Kg (Antisedan, Orion Pharma, Milan, Italy), Flumazenil 0.5 mg/Kg (Anexate flumazenil, Roche, Basel, Switzerland), Buprenorphine 0.1 mg/Kg (Temgesic buprenorphinum, Schering-Plough, NJ, USA) was delivered subcutaneously and 500 μl of intraperitoneal sterile saline was injected for rehydration. Animals were then placed on a heating pad set to 37°C with food and water *ad libitum*. Animals were returned to the home cage when diuresis and gastrointestinal transit had restarted and signs of acute pain were absent. Anesthesia for the entire surgery in general lasted 1 h–1 h 30.

**FIGURE 1:**
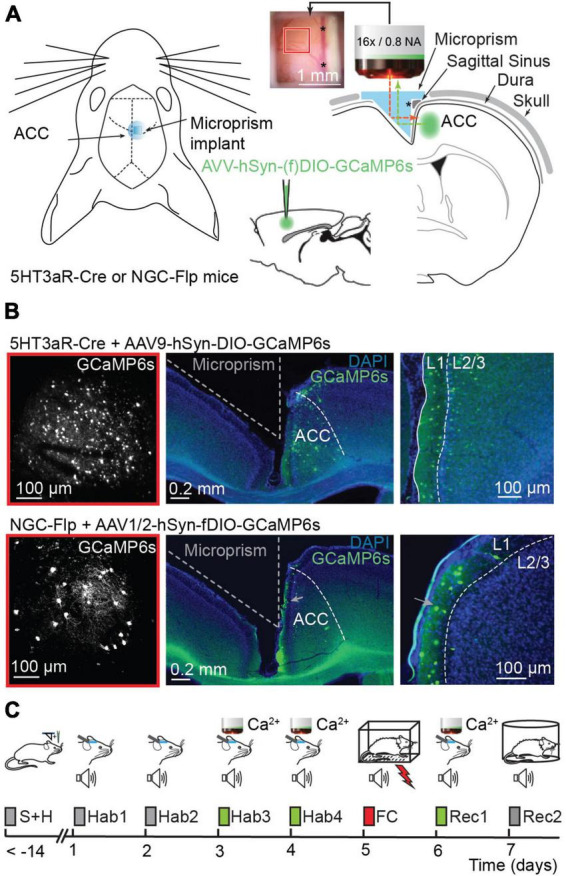
Calcium imaging in the ACC before and after fear conditioning. **(A)** Left, schematic representation of prism implantation in 5HT3aR-Cre or NGC-Flp mice. Right, AAV9-hSyn-DIO.GCaMP6s or AAV1/2-hSyn-fDIO.GCaMP6s viral vectors were injected in the ACC of 5HT3aR-Cre or NGC-Flp mice, respectively. Immediately after the injection, a microprism was implanted in the contralateral hemisphere. The inset shows an example of the cranial window with the underlying cortex and vasculature 2 weeks after surgery. The red and green dotted lines represent the excitation beam and the emitted light path, respectively, through the implanted prism. Note that the reflective hypotenuse of the prism converts the horizontal plane into a vertical imaging plane. The asterisk indicates the sagittal sinus, which is also visible on the right-hand side of the inset. **(B)** Left, example of the field of view with 2PLSM in GCaMP6-expressing 5HT3aR-Cre (top) or in NGC-Flp (bottom) mice. The red square compares the field of view to the size of the cranial window in panel **(A)**. Right, representative *post hoc* immunostaining showing GCaMP6s expression (green) in the ACC. The integrity of the targeted cortex for imaging remains intact. In blue, DAPI-staining. **(C)** The FC paradigm. After surgery, mice are handled daily for at least 7 days. The experimental paradigm starts with 4 days of habituation (Hab1-4) where the mice are head-fixed and exposed to tones. In the last 2 days of habituation (Hab-3 and Hab-4) this is combined with 2PLSM. On day 5, the mice are subjected to fear conditioning (FC) in a conditioning box while freely moving. 24 h later a recall session (Rec-1) is performed while mice are head-fixed and imaged. On day 7, animals undergo a second recall session (Rec-2) while freely moving, allowing the scoring of freezing behavior.

Microprism surgical implantation was performed based on a modified protocol described previously (Low et al., 2014). Specifically, 1.5 mm sized microprisms with aluminum coating on the hypotenuse (BK7 glass, TowerOptical, CT, USA, MPCH-1.5) were used. These 1.5 mm microprisms were inserted in the interhemispheric fissure, in the subdural space of the left hemisphere, with the lateral face flush with the dural falx and facing the right ACC. In this configuration, the excitation light entered the microprism through the top face and the emitted fluorescence light through the lateral face (Figure 1A). Both were reflected on the aluminum-coated hypotenuse. Before surgery, the microprisms and coverslips were cleaned using sterile saline and dried with microscope paper (Kimtech 7552) to reduce dust layers. Under a surgery stereo microscope (M80, Leica, Wetzlar, Germany) the microprisms were then bonded to a 3 mm coverslip (64-0720, Multi-channel systems) using one drop of UV-curing glue (Norland, PA, USA) which was placed with the tip of a needle on the center of the window. Care was taken to have the glue evenly distributed over the entire surface of the prisms to avoid unequal transparencies. Curing was performed using a UVA lamp. Surgical loop was used (M80, Leica, Wetzlar, Germany) and surgical field illumination was done using fiber lights (MI-150, Dolan-Jenner). The craniotomy was performed using a 0.6 mm dental drill (HM1 006, Meisinger, Düsseldorf, Germany). Sterile cortex buffer [125 mM NaCl (7.21 g NaCl), 5 mM KCl (0.372 g KCl), 10 mM glucose (1.802 g glucose), 10 mM HEPES (2.38 g HEPES), 2 mM CaCl2 (2 ml 1M CaCl2) and 2 mM MgSO4 (2 ml 1M MgSO4) in distilled 1l H2O] to moisturize the tissue. For hemostasis, small pieces of sterilized microscope paper were combined with humidified Gelfoam (Pfizer, New York, NY, USA). A surgical tray sterilized with ethanol 70% was used to contain the following micro instruments during the procedure: Vannas Spring Scissors (15070-08, Fine Science Tools, Heidelberg, Germany), Extra Fine Bonn Scissors (14083-08, Fine Science Tools, Heidelberg, Germany), two Student Dumont #5 Forceps (91150-20, Fine Science Tools, Heidelberg, Germany) angle sharp microtool (Ergobrobe 202660, M + W dental), two standard blue forceps (DumontAA 11210-10, Fine Science Tools, Heidelberg, Germany, microblade), microspatula (orban scaler, World Precision Instrument, FL, USA), 3 mm disposable biopsy puncher (801818, World Precision Instrument), two plastic forceps (11700-00, Fine Science Tools, Heidelberg, Germany), and a skin tissue forceps (Adson 14226-G, World Precision Instrument, FL, USA). The window was sealed using acrylic superglue (110-41-180, Patex), and a head cap was created out of dental acrylic (Jet repair, Lang Dental, NY, USA), which was adhered to the skull by applying the liquid mix to the superglue-covered skull surface. The aluminum holders (1269-7475-001, Protolab, MN, USA) were fixed to the head cap by using another layer of dental acrylic.

Stereotaxic injections of adeno-associated viral (AAV) vectors were performed in the contralateral, intact hemisphere right after the craniotomy and before microprism implantation. Injection pipettes were made from Drummond Wiretrol II 1–5 μl capillaries (DRUM5-000-2005, Drummond). Stereotaxic injections were delivered to the ACC through two separate injection points on the rostro-caudal axis in order to avoid tissue trauma-related gliosis in the center of the future imaged area. The ACC spans a cortical area extending from the caudal edge of the PL cortex to the rostral edge of the retrosplenial cortex (RSP). The surface vasculature over this region is dense and the size of the sagittal sinus greatly varies in different mice. To avoid penetrating the vasculature, the injection slightly varied between mice along the rosto-caudal axis, but always well within the range of the ACC. The injection coordinates were the following: rostro-caudal, between +1.5 mm and −0.7 mm around bregma; dorso-ventral between 0.7 and 0.8 mm from the pia; medio-lateral, between 0.2 and 0.3 mm from the sagittal suture. Therefore, all the injections were centered in the dorsal part of the ACC, centered in the Cg1 subregion as defined by Paxinos atlas (or the ACAd according to the Allen Brain Atlas), which aligns with the well characterized parcellation of the cingulate cortex in rodents (van Heukelum et al., 2020). Expression of GCaMP6s was allowed to ramp up for at least 2–3 weeks after surgery. Imaging was started if the brain’s vasculature and surface maintained a clear and healthy appearance 2–3 weeks after the microprism implantation. The intradural and subdural and intracortical vasculature was used to register regions of interest and to re-identify neurons at different time points.

### Fear conditioning protocol

Mice were handled for at least 7 days (10 min, twice a day) to allow familiarization with the experimenter and microscope enclosure. Subsequently, mice underwent four habituation sessions (Hab1-4, 1 session/day), during which they were awake and head-fixed in the microscope setup and exposed to the conditioning tones (conditioned stimulus, CS). This protocol was composed of a 5-min silent baseline period followed by 10 tones (70 dB, 7.5 kHz, 200 ms duration) delivered at 1 Hz for 30 s (Grewe et al., 2017) with variable inter-stimulus intervals (ISI) of 60–90 s. Neuronal calcium signals in the ACC were imaged during the last 2 days of habituation (Hab-3 and Hab-4). On day 5, mice underwent a cued fear conditioning (FC) protocol while freely moving in a different context, which consisted of a 25 cm × 25 cm × 35 cm custom made plexiglass enclosure built around a stainless-steel electrified grid placed in a custom made soundproof 50 cm × 50 cm × 50 cm box equipped with two speakers and LED lighting (100 Lux). The conditioning protocol was run by ANY-maze software (Stoelting Europe), which paired a series of 5 tones (70 dB, 7.5 kHz freq, 200 ms duration delivered at 1 Hz for 30 s) with an electric shock (the unconditioned stimulus, US; 0.6 mA). The shock was delivered in the last 2 s of each tone period. The inter-stimulus intervals randomly varied from 60 to 90 s. After conditioning, the animals were returned to the home cage. The conditioning box was cleaned with 70% ethanol before and after each session. Animals were monitored using a camera positioned above the conditioning enclosure. Freezing was scored automatically by the ANY-maze software using a 2-s threshold for the absence of movement (based on pixel variance detection). As controls, a different set of mice underwent a pseudo-conditioning protocol in which the US was randomly given during the ISI following the CS. On day 6, conditioned mice were again head-fixed in the microscope setup and tested for fear memory. This memory recall session (Rec-1) was performed similarly to Hab-3 and Hab-4, i.e., calcium signals were imaged while presenting 10× the CS with randomly varied ISI durations (60–90 s). 24 h after Rec-1, a second recall session (Rec-2) was performed while mice were freely moving. The mice were placed in a novel chamber, exposed to tones, and freezing was evaluated as described for the FC session.

All statistical analyses were performed using R-Studio and GraphPad Prism. Significance levels were denoted as *p < 0.05, ^**^p < 0.01, ^***^p < 0.001, ^****^p < 0.0001. No statistical calculation was made to estimate sample sizes. Comparison tests were performed as two-sided.

### *Post hoc* histology and immunostaining

For regular *post hoc* assessment, mice were anaesthetized with 3–4% isoflurane (mixed with O_2_) induction followed by an intraperitoneal injection of Pentobarbital (150 mg/Kg, Esconarkon ad us. Vet. Streuli, Uznach, Switzerland) combined with subcutaneous injection of buprenorphine (0.1 mg/kg, Temgesic, Schering-Plough, NJ, USA) analgesia. After reaching deep anesthesia checked by toe pinching, before reaching cardiorespiratory arrest, transcardiac perfusion (4% paraformaldehyde (PFA), 1× PBS, pH 7.4) was performed. The brains were extracted and post-fixed overnight at 4°C in 4% PFA and subsequently transferred in PBS. *Post hoc* 50–100 μm-thick axial coronal sections were produced using a vibratome (Leica VT 1000, Germany) and stored in PBS 0.1% Na-azide. The sections were imaged using a Zeiss Confocal LSM800 Airyscan (Axio Imager.Z2 Basis LSM 800 microscope).

For the immunohistological characterization the NGC-Flp mouse line, mice (*n* = 3) received an injection of AAV1/2-hSyn.fDIO.GCaMP6s as above, but without a microprism implant. 20 days post-surgery they were anaesthetized and fixed as above. The brains were collected and left in 4% PFA 3h at RT for further fixation. Coronal brain sections (40 μm thickness) were produced using a vibratome (Leica VT1200S; Leica Microsystems). First, the sections were pre-incubated in PBS 1% BSA, 0.3% Triton X-100 for 1 h at room temperature. Then, the following primary antibodies were used: mouse anti-GFP 1:500 (Abcam, Cambridge, United Kingdom, #ab1218), rabbit anti-VIP 1:500 (Immunostar, WI, USA, #20077), rabbit anti-SST 1:1000 (Invitrogen, MA, USA, #PA5-82678), goat anti-PV 1:2000 (Swant, Burgdorf, Switzerland, #PVG-214) diluted in the pre-incubation solution and applied for overnight at 4°C. As secondary antibodies we used Alexa anti-mouse 488 (1:1000); anti-rabbit 568 (1:1000); anti-goat 568 (1:1000) diluted in PBS 1%BSA and applied for 1 h in the dark at room temperature. Finally, for cell nuclei staining, Hoechst 1:5000 (Invitrogen, MA, USA, #H3570) was applied for 20 min in the dark at room temperature. Images were taken using a Leica Stellaris confocal microscope 20× objective. Image analysis on these images was performed in Fiji (NIH, Bethesda). Regions of interest (ROI) contours were drawn around neuronal somata, and the mean pixel value at the focal plane with the highest value for each cell was measured. PV, SST, and VIP antibody-stained (red) cells and GFP antibody-stained (green) cells were selected, as well as red cells in an area outside of the injection zone (controls for GFP-background). Number of analyzed cells: VIP: 297; SST: 316; PV: 539.

### 2-photon laser scanning microscopy

For the detection of calcium signals, mice were head-fixed under the microscope and imaged while awake. We used a custom-built 2-photon laser scanning microscope [2PLSM; (Holtmaat et al., 2009)], equipped with a 16 × 0.8NA water immersion-objective (Nikon, CFI75), and controlled with Scanimage 2016b.^4^ Fluorophores were excited using a Ti: Sapphire laser (Chameleon Ultra II, Coherent, CA, USA) tuned to λ = 980 m. Fluorescent signals were collected with GaAsP photomultiplier tubes (10770PB-40, Hamamatsu, Japan). GCaMP6s signals were collected through a dichroic mirror (565dcxr, Chroma) and emission filter (ET525/50 m, Chroma). Images were acquired at a 8 Hz-scanning rate, and each image consisted of a single plane (723 × 675 μm; 512 × 256 pixels). The imaging plane was between 10–100 μm below the pia and therefore well within L1.

### Image processing and calcium signal analysis

Images were processed using custom-written MATLAB (Mathworks, MA, USA) script and ImageJ (NIH, Bethesda, MD, USA). Lateral and axial motion errors were corrected using the ImageJ plugin Stack Aligner by performing a rigid image registration across all frames of the movie. To extract the GCaMP6s fluorescence signals from individual neurons, regions of interest (ROIs) were drawn manually for each session. The fluorescence time-course of each neuron was measured as the average of all pixel values of the GCaMP6s signal within the ROI. Calcium activity was then evaluated as ΔF/F, where F is the lowest 30th percentile mean intensity of all the trace. ΔF/F signals of all cells were subsequently log-transformed with the formula $v_{i}^{\prime }=\log_{2}\left(\max\left(0,v_{i}\right)+1\right)$. For each tone, the obtained signals were averaged over 10-s periods: 20–10 s pre-tone (ISI), 10–0 s pre-tone (ISI), and 0–10 s from tone onset (Tone).

#### Cell classification and exclusion

**FIGURE 2:**
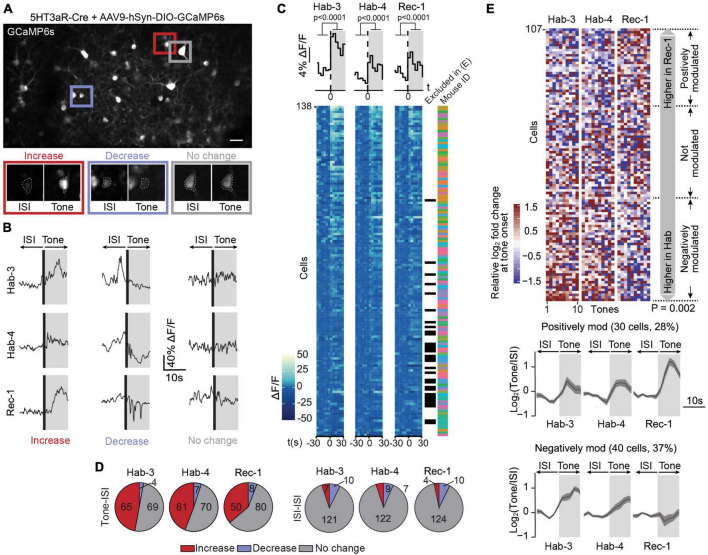
Calcium signals in 5HT3aR interneurons are bimodally modulated following fear conditioning. **(A)** A field of view containing GCaMP6s-expressing 5HT3aR neurons in L1 of ACC (scale bar: 50 μm). The image is an average projection of the full-length imaging period. The insets show examples of cells displaying an increase, decrease or no change in calcium signals at tone onset. Insets were generated from the average projection of the first 10 s of tone and the last 10 s of the preceding ISI. **(B)** Example ΔF/F traces of individual neurons over a 20-s window spanning one ISI-tone transition in each imaging session (Hab-3, Hab-4, Rec-1). **(C)** Tone-evoked 5HT3aR neuron activity. The heatmaps represent calcium signals (ΔF/F) in neurons before and after tone onset. Each row represents a single cell. Each square represents a 5-s bin averaged across all 10 tone presentations. Cells are sorted according to their responsiveness in Hab3, with the highest responses on top. Above the heatmaps, the grand average ΔF/F during tone vs. ISI (mean activity during first and last 30 s, *t*-test *P* < 0.0001, *n* = 138). Next to the heatmaps, the black checkmarks indicate cells that did not significantly respond in any of the recording sessions and were therefore excluded from further analysis (31). The color-coded checkmarks identify the mouse from which the recorded cell was derived. **(D)** Fraction of cells that increase, decrease, or do not show changes in activity at tone presentation (left) or during ISIs (right) for each recording session. **(E)** Top, heatmap of the 107 responding cells (8 mice) sorted according to the difference of the median response to tones in Rec-1 relative to Hab-3 and Hab-4. Each square represents the relative log_2_-fold change of the calcium signal at tone onset. Margin analysis revealed two groups of neurons whose activity is dynamically modulated following fear learning (random permutation analysis *P* = 0.002). A total of 30 cells are positively modulated by fear learning (median activity difference > 0.3) and 40 cells are negatively modulated (median activity difference < –0.3). Bottom, for both groups of neurons, the lower panel shows the average calcium trace (with SEM) of the tone-evoked calcium signals during all three imaging sessions. Gray areas represent the 10-s tone duration.

**FIGURE 3:**
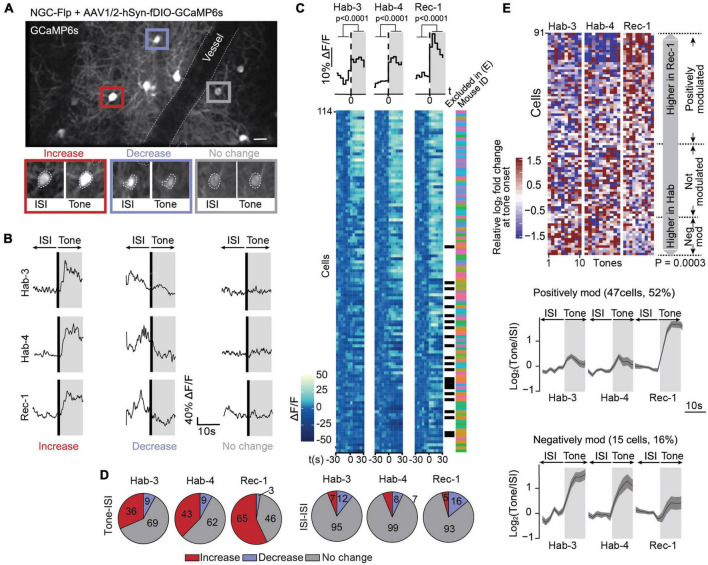
Calcium signals in NGCs are mostly positively modulated following fear conditioning. **(A)** A field of view containing GCaMP6s-expressing NGCs in L1 of the ACC (scale bar: 50 μm). This image is an average projection of the full-length imaging period. The insets show examples of cells displaying an increase, decrease or no change in calcium signals at tone onset. Insets were generated from the average projection of the first 10 s of tone and the last 10 s of the preceding ISI. **(B)** Example ΔF/F traces of individual neurons over a 20-s window spanning one ISI-tone transition in each imaging session (Hab-3, Hab-4, Rec-1). **(C)** Tone-evoked NGC activity. The heatmaps represent calcium signals (ΔF/F) in neurons before and after tone onset. Each row represents a single cell. Each square represents a 5-s bin averaged across all 10 tone presentations. Cells are sorted according to their responsiveness in Hab-3, with the highest responses on top. Above the heatmaps, the grand average ΔF/F during tone vs. ISI (mean activity during first and last 30 s, *t*-test *P* < 0.0001, *n* = 114). Next to the heatmaps, the black checkmarks indicate cells that did not significantly respond in any of the recording sessions and were therefore excluded from further analysis (23). The color-coded checkmarks identify the mouse from which the recorded cell was derived. **(D)** Fraction of cells that increase, decrease, or do not show changes in activity at tone presentation (left) or during ISIs (right) for each recording session. **(E)** Top, heatmap of the 91 responding cells (9 mice) sorted according to the difference of the median response to tones in Rec-1 relative to Hab-3 and Hab-4. Each square represents the relative log_2_-fold change of the calcium signal at tone onset. Margin analysis revealed two groups of neurons whose activity is dynamically modulated following fear learning (random permutation analysis *P* = 0.0003). A total of 47 cells are positively modulated by fear learning (median activity difference > 0.3) and 15 cells are negatively modulated (median activity difference < –0.3). Bottom, for both groups of neurons, the lower panel shows the average calcium trace (with SEM) of the tone-evoked calcium signals during all three imaging sessions. Gray areas represent the 10-s tone duration.

We generated scatterplots of the 10 s-average-signals to visually inspect the difference between the signal during tones (y-axis: signal average of 0–10 s from tone onset) with the signal during the preceding ISI (x-axis: signal average of 10–0 s pre-tone). Because of the above log-transformation, the distance to the diagonal on this plot is an estimate of the log_2_ (“fold-change”) during the tone. A tone-associated response was considered significant if it had a log_2_ (“fold-change”) distance to the diagonal higher than a predetermined threshold (see below). Based on this, neurons were subdivided into three classes: increase–cells displaying a log_2_ (“fold-change”) increase above the threshold in at least 6 out of the 10 tones within a session; decrease–cells displaying a log_2_ (“fold-change”) decrease above the threshold in at least 6 out of the 10 tones within a session, no change–all remaining cells. A cell was considered non-responsive to tones if it belonged to the “no change” class in all recording sessions (Hab-3, Hab-4, Rec-1) (indicated in black in Figures 2C, 3C). These cells were excluded from cell activity change enrichment test. The threshold was independently determined for each group of mice (NGC, 5HT3aR, pseudo-conditioned 5HT3aR), and set such that it excluded 33% of the cells when only habituation sessions (Hab-3, Hab-4) were considered.

To verify the specificity of this approach, the same analysis was performed to estimate the log_2_ (“fold-change”) between two ISI periods (i.e., comparing the average-signals over 20–10 s pre-tone with the average-signals over 10–0 s pre-tone).

#### Cell activity change enrichment test

To test whether there was a significant change in tone responses between habituation and recall sessions, we first generated a heatmap with the log_2_ (”fold-changes”) of each cell for the 30 tones of all sessions (Hab-3, Hab-4, Rec-1) (Figures 2E, 3E). In this heatmap, cells were sorted according to the difference in the signal between the habituation and recall sessions (detail of the criterion used for sorting in next section). The color of each square is the log_2_ fold-change between the first 10 s of the tone vs. the last 10 s of the ISI. The colors are saturated, with fold-changes above 1.5 (resp. below −1.5) being assigned the 1.5 color (resp. −1.5 color). A cutoff was chosen (see below) to classify a cell as ‘modulated’ or ‘not modulated’ between habituation and recall days, and we counted the number of cells satisfying this criterion. Subsequently, a simulated dataset of 10^4^ cells was generated by randomly selecting cells from the initial dataset and randomly permuting their responses to 30 tones. Then, the same criterion was applied as above, and we performed a binomial test to assert that the number of cells changing their activity in the real dataset was higher than the amount in the simulated dataset.

#### Cell activity change criterion

The criterion that was used to sort the heatmap and classify a cell as modulated or not between habituation and recall conditions is the difference of the medians, slightly adapted to be more rigid and so that it defines three groups of cells: increasing, decreasing, no-change. The exact formula of our sorting criteria is:


$$\delta_{\text{i}}=\text{max}\,\left(0,5^{t\,h}\,\left(r_{i}\right)+9^{t\,h}\,\left(-h_{i}\right)\right)-\text{max}\,\left(0,9^{t\,h}\,\left(h_{i}\right)+5^{t\,h}\,\left(-r_{i}\right)\right)$$

where:

n*^th^* (x) is the n*^th^* smallest value of vector x.

r_*i*_ (resp. h_*i*_) is the vector of fold-changes of the i*^th^* cell, at the 10 (resp. 20) tones during recall (resp. habituation).

In other words, δ_*i*_ is:

positive if the i*^th^* cell has an activity that is higher in 6 recall tones than in 12 habituation tones.

negative if the i*^th^* cell has an activity that is smaller in 6 recall tones than in 12 habituation tones.

zero otherwise

In our enrichment test, a cell was considered to have changed its response between habituation and recall if δ_*i*_ was not zero.

## Results

### Labeling and imaging of ACC L1 interneurons in a fear conditioning paradigm

In order to investigate neuronal activity of ACC L1 interneurons, we expressed the genetically-encoded calcium indicator GCaMP6s using recombinase-dependent AAV vectors in two different genetically modified mouse lines. In a first approach, the vectors were injected in 5HT3aR-Cre mice that constitutively express Cre in a heterogenous population of interneurons in L1, albeit a more precise functional characterization is gradually building (Lee et al., 2010; Gerfen et al., 2013; Takesian et al., 2018; Gouwens et al., 2020; Anastasiades and Carter, 2021). Hence, we will refer to the cells that are labeled using 5HT3aR-Cre mice in combination with AAV vectors as 5HT3aR neurons. In a second set of experiments, we specifically targeted NGCs which form a distinct subgroup of interneurons in L1 that largely belong to 5HT3aR-Cre neuron population (Niquille et al., 2018; Tasic et al., 2018; Gouwens et al., 2020; Ibrahim et al., 2021). To accomplish this, we took advantage of an Hmx3-Cre mouse line, a reporter line previously shown to fate map NGCs by targeting cortical interneurons originating from the preoptic area (Gelman et al., 2009; Niquille et al., 2018). Since in this line Cre is only expressed at embryonic stages, we crossed it with a LSL-Flp mouse line that harbors a Cre-conditional Rosa-26-CAG-LSL-Flp allele (Gelman et al., 2009; Niquille et al., 2018). The offspring of this crossing transiently expresses Cre in NGC precursors, which subsequently switches on the constitutive Rosa-26-CAG-driven expression of Flp recombinase in the mature population of NGCs (Supplementary Figure 1A). This offspring, which we hereafter refer to as NGC-Flp mice, allowed targeted expression of GCaMP6s in NGCs in the adult brain using Flp dependent AAV vectors. The characteristics of the labeled cells indeed strongly resembled those of NGCs, i.e., they were enriched in L1, had an elongated morphology, and projected thin axons with elaborate branches (Supplementary Figures 1B, C). The presumptive NGC identity in these mice was further confirmed using an immunolabeling of interneuron molecular markers. Due to the absence of a general molecular marker of NGCs, sections were labeled using antibodies for three other main classes of interneurons: PV, SST, and VIP. The absence of co-labeling of any of these markers indicates that the GCaMP6s-expressing cells were molecularly distinct from the PV, SST, and VIP interneuron population (Supplementary Figure 1D), further corroborating their putative NGC identity (Jiang et al., 2013; Lee et al., 2015; Overstreet-Wadiche and McBain, 2015; Niquille et al., 2018; Schuman et al., 2019; Gouwens et al., 2020).

To longitudinally track L1 interneuron activity, we implanted a microprism into the interhemispheric longitudinal fissure and used 2PLSM in head-fixed awake mice (Low et al., 2014; Figure 1A). *Post hoc* assessment of microprism-implanted brains confirmed that GCaMP6s expressing neurons were abundantly present in L1 in both 5HT3aR-Cre and NGC-Flp mice (Figure 1B). The labeled NGC population showed a strong enrichment in L1 relative to the other cortical layers, in accordance with previous findings (Jiang et al., 2015; Tremblay et al., 2016; Niquille et al., 2018). The *post hoc* assessment also indicated that the microprism implant had left the microanatomy and layers of the injected right hemisphere intact (Figure 1B).

Once mice were recovered from surgery (>7 days), they were handled for 7 days and then habituated for 4 days (Hab-1-4; 1 session/day) to the head fixation in the microscope during which they were exposed to 10 tones (10× CS) per session without shocks (US) (Figure 1C; Supplementary Figure 2A). The fear conditioning paradigm (FC; 5× CS-US pairing) was performed on freely moving mice in a different environment. The subsequent memory recall session (Rec-1; 10× CS) was again performed while mice were head-fixed under the microscope. A second recall session (Rec-2) was performed in freely moving mice in a further different context to assess the CS-US associative memory using the freezing time as a measure. Calcium signals were imaged in the last two habituation sessions (Hab-3 and 4) as well as in the first recall session (Rec-1) (Figure 1C).

During the FC session, the average duration of freezing upon tone presentation increased starting after the second CS-US pairing and was significantly higher by the fifth tone (Supplementary Figure 2B). When mice were re-exposed to the CS in Rec-2, they displayed a selective increase in conditioned freezing (Supplementary Figure 2C) and the level of freezing remained high for the whole duration of the protocol (Supplementary Figures 2C, D). Mice subjected to pseudo-conditioning, in which the CS and US were not paired (Supplementary Figure 2A), tended to gradually increase the duration of freezing during tone presentation, even if such increase was not significant (Supplementary Figure 2E). Most importantly, pseudo-conditioned mice did not display increased freezing in Rec-2, indicating that they had not produced a CS-US associative memory (Supplementary Figures 2F, G).

NGC-Flp mice showed a similar behavioral response to FC as the 5HT3aR-Cre mice, i.e., they started to increase freezing duration after the second CS-US pairing (Supplementary Figure 2H), and they had significantly longer freezing durations in Rec-2 (Supplementary Figures 2I, J).

Together, these data show that the fear conditioning protocol created a specific and robust CS-US associative memory, which lasted throughout and beyond the Rec-1 imaging session. Therefore, by comparing the calcium signal dynamics during Hab-3, Hab-4, and Rec-1 in the conditioned versus the pseudo-conditioned mice, we were able to identify the interneurons that are putatively involved in the successful formation or recall of a CS-US associative memory.

### Tone-evoked calcium signals in 5HT3aR neurons are bimodally modulated by fear conditioning

In order to investigate the evolution of L1 5HT3aR interneuron activity in response to conditioned tones during fear learning, we longitudinally recorded calcium signals in 138 cells from eight 5HT3aR-Cre mice during three sessions (Hab-3, Hab-4, and Rec-1; Supplementary Figure 3A). Neurons displayed heterogeneous responses across all sessions, including increases, decreases and no change in calcium signals upon tone presentations (Figures 2B, C). On average the calcium signals increased upon tones, indicating that a substantial fraction of this population of L1 ACC neurons responds to auditory stimuli (Figure 2C). To further classify individual responses, we averaged for each cell the ΔF/F over a 10-s period immediately before and after tone onset and calculated the post-pre log_2_-fold change (see Section “Materials and methods”). In all sessions we found a substantial fraction of neurons that increased their activity upon tone presentations while only a few did not change or decrease their activity (Figure 2D) indicating that 5HT3aR interneuron activity can be evoked by tones. When the same classification was performed on the post-pre log_2_-fold change between 10 s periods outside tones, most cells showed no activity change, confirming that the responses were tone-specific (Figure 2D). Based on this analysis, we found 31 out of 138 cells that showed no response in any session. They were excluded for further analysis (see Section “Materials and methods”). To assess whether tone-responsive cells dynamically modified their activity following CS-US association, we compared their responses during Hab-3 + 4 to the responses in Rec-1. First, we sorted the 107 responding cells according to the difference in their tone responses between habituation and recall (Figure 2E). In order to assess if FC affects those responses, we performed margin analysis to find clusters of differentially modulated neurons. We obtained three groups of neurons: a positively modulated cluster (30 cells; 30%), in which 60% of the tone responses during Rec-1 (i.e., 6 out of 10 tones) are higher than 60% of the tone responses during Hab-3 and 4 (i.e., 12 of 20 tones); a negatively modulated cluster (40 cells; 37%) where 60% of the tone response during Rec-1 are smaller than 60% of the tone responses during Hab-3 and 4; and a third non-modulated cluster of the remaining cells (Figure 2E). All clusters were populated by neurons obtained from different mice (Supplementary Figure 4A), confirming that these types of modulation are transversally present. Importantly, random permutation analysis confirmed the significant separation of the obtained neuronal clusters (positively or negatively modulated neurons following fear conditioning; *P* = 0.002). These results demonstrate that 67% of tone-responding ACC L1 5HT3aR cells are bimodally modulated by fear learning. To verify whether this modulation was specifically induced by the CS following FC, we performed the same margin and permutation analysis using the calcium signals observed in between tone presentations [two periods of 10 s each within the inter-stimulus interval (ISI)]. ISI analysis did not reveal any clusters (*P* = 0.778), indicating that FC does not cause a generalized (CS-unrelated) modulation of activity in these cells (Supplementary Figure 4B).

To validate that the modulation of the responses is specifically associated with FC, we performed the same analysis in the pseudo-conditioned mice (6 mice, 111 cells; Supplementary Figure 3B). Again, results did not reveal any positively or negatively modulated clusters (88 responsive cells, *P* = 0.301; Supplementary Figure 4C). Similarly, no clusters were found when activity in 10-s ISIs were considered in pseudo-conditioned mice (*P* = 0.900; Supplementary Figure 4D).

Overall, these data demonstrate that 5HT3aR ACC L1 INs can selectively respond to auditory stimuli and these responses are bidirectionally modulated by FC.

### Tone-evoked responses of NGCs increase upon fear conditioning

Similar to the global 5HT3aR neuron population, NGCs recorded during different days (Supplementary Figure 3C) displayed heterogeneous responses upon CS presentation, i.e., cells either increased, decreased or showed no change in calcium signals during tones across sessions (Hab-3, 4 and Rec-1; Figures 3A, B). At the population level, NGCs also showed a marked tone-evoked increase in activity across all sessions (Figure 3C). Single cell analysis revealed that a substantial fraction of neurons increased their activity while only a few decreased their activity (Figure 3D). When the same classification analysis was performed on 10-s periods during ISIs, most cells showed no change in activity, confirming the tone-specificity of the responses (Figure 3D). From the 114 recorded cells (9 mice), 91 (80%) responders were kept for further analysis (see Section “Materials and methods”). As before, we compared the neurons’ responses during Hab-3 + 4 to those in Rec-1 to assess whether FC had a modulating effect on tone-evoked responses in NGCs. Cells were sorted according to the difference in responses between habituation and recall (Figure 3E). Margin and random permutation analysis (*P* = 0.0003) revealed the emergence of three clusters of FC-modulated responses. Differently to what we observed for the 5HT3aR neuronal population, NGCs were enriched in positively modulated cells (47/91; 52%), with only a small fraction decreasing CS-evoked responses following FC (15/91; 16%) (Figure 3E). The positively modulated cluster contained cells from all mice, the negatively modulated cluster did not (Supplementary Figure 5A). Like what was observed for the 5HT3aR population, no clusters were detected when analyzing 10-s segments of ISIs in the same group of cells (*P* = 0.958; Supplementary Figure 5B), confirming that FC had specifically modulated the tone-evoked responsiveness in NGCs.

**FIGURE 4:**
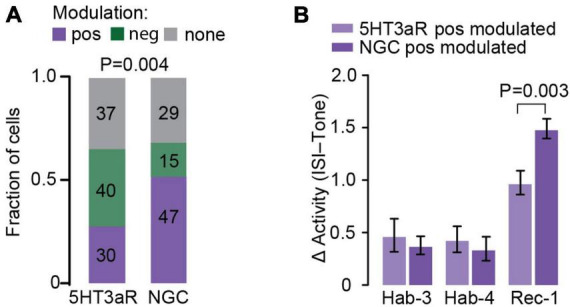
Comparison of 5HT3aR neurons and NGC activity upon FC. **(A)** Fraction of the cells whose activity is positively, negatively or not modulated following fear learning in the 5HT3aR and NGC populations. The number of neurons populating each cluster is indicated inside each color-coded box. Positively modulated neurons are enriched in the NGC population (Chi square test; *P* = 0.004). **(B)** Direct comparison of tone-evoked responses between positively 5HT3aR neurons and NGCs. Δ activity was calculated for each cell as an average across all tones of the difference in the log_2_-fold change during the first 10s of CS and the log_2_-fold change during the last 10s of ISI. While the average tone-evoked response in habituation sessions does not differ between both groups, it is higher in NGC population during Rec-1 [RM-Two-Way ANOVA, *F*(2,150) = 6.681, *P* = 0.003; *Post hoc* analysis Sidak, *N* = 30–47 cells].

The positively modulated NGCs did not only constitute a larger fraction of the population as compared to those of the 5HT3aR population (52 vs. 28%; Figure 4A), they also showed larger calcium signals during Rec-1 (no difference was observed for Hab-3 and 4; Figure 4B). Conversely, negatively modulated neurons were less abundant within the NGC population as compared to the 5HT3aR population (16 vs. 37%; Figure 4A), suggesting that NGCs may represent a functionally distinct type when compared to 5HT3aR L1 INs. Together, the data suggest that interneuron subpopulations in L1 of ACC may differentially contribute to FC memory formation and recall.

## Discussion

We repeatedly imaged calcium signals in two L1 interneuron subtypes of the ACC in a mouse model for fear learning. We observed that tones (CS) evoked activity in ∼80% of the L1 5HT3aR cells and NGCs before and after the conditioning protocol. Of these tone responsive populations, ∼70% of the total responsive neurons were bidirectionally modulated by FC, i.e., some cells increased and others decreased their responses to the CS upon fear learning. The NGC population was relatively enriched in positively modulated neurons. Altogether, the data indicate that auditory stimuli evoke responses in subpopulations of ACC L1 INs, which can be modified by fear learning. In particular, our data show that fear learning is associated with a net increase in the activity of an inhibitory motif constituted of NGCs which are known to target pyramidal cell apical dendrites and to be targeted by long-range excitatory inputs (Muralidhar et al., 2013; Hou and Capogna, 2018). Together, this suggests that the integration of sensory inputs and/or executive outputs of the ACC are modulated by fear learning. This may have important implications for the understanding of how aversive memories are encoded and stored in cortical circuits.

### Tone-evoked responses in ACC

The first remarkable observation provided by this work is the strong activation of subpopulations of L1 interneurons upon the presentation of the auditory CS, even before learning (Figures 2B–D, 3B–D). Previous studies have shown that the ACC can be activated by visual and somatosensory inputs, in particular when they are noxious (Johansen et al., 2001; Wei and Zhuo, 2001; Blom et al., 2014; Ma et al., 2016; Sidorov et al., 2020). Such responses may originate from afferents of the medial thalamus (MT) (Hsu and Shyu, 1997; Hsu et al., 2000), or somatosensory and visual cortices (Sidorov et al., 2020; Singh et al., 2020).

Our data suggest that auditory information is processed by the ACC as well. Auditory signals may also derive from thalamic and cortical sensory areas. However, the responses may not necessarily represent low-order inputs from the auditory stream to the ACC, but rather be the result of high-order processing of the sound as a contextual stimulus. In addition, it is also possible that tone-evoked activity that we observed did not merely represent the perceptual processing of auditory input *per se* but rather a sound-evoked increase in attention levels, known to increase and depend on activity in the ACC (Petersen and Posner, 2012; Wu et al., 2017).

Since the auditory stimulus in our paradigm was delivered as pips for a period of 30 s, the temporal relationship between the sound and neuronal activity was difficult to disentangle. To circumvent this, we focused our analysis around tone onset, and assessed how this was modulated by fear learning.

### Bidirectional modulation of responses upon fear learning

The second main observation was the bidirectional modulation of the CS-evoked responses in subpopulations of the L1 interneurons (Figures 2, 3). We demonstrated that this modulation is a specific learning effect. First, because a pseudo-conditioning protocol in which mice received shocks in a dissociated manner from the CS did not result in significant CS-evoked response alterations (Supplementary Figure 4); and second because spontaneous calcium signals during ISIs were not changed (Supplementary Figure 4). A bimodal response in fear learning-related activity is not unique to the ACC. Our results align with observations in L1 interneurons of the auditory cortex (Letzkus et al., 2011; Abs et al., 2018), pyramidal neurons in the amygdala (Grewe et al., 2017) and PV neurons in the dmPFC (Courtin et al., 2014).

In addition, activity of the ACC has been reported in association with various subcomponents of fear learning (see for reviews: Jovanovic and Norrholm, 2011; Hinojosa et al., 2019). It has been implicated in storage of fear memories (Descalzi et al., 2012; Einarsson et al., 2015), but also in fear termination (Steenland et al., 2012; de Lima et al., 2022) and resistance to extinction (Louzolo et al., 2022). Taken together, these findings suggest that specific subpopulations of projecting neurons in the ACC may be bimodally regulated by fear learning, allowing targeted facilitation of fear-encoding neurons and a suppression of antagonistic neuronal subsets. Such bimodal regulation may be achieved by inhibition derived from either up-regulated or down-regulated L1 interneuron activity, which may be directed to different subsets of principal neurons.

### The role of L1 interneurons subtypes

Using the intersectional genetic strategy described in the Results, we were able to specifically monitor the activity of the NGC subpopulation of L1 5HT3aR interneurons. NGCs are *Hmx3*-derived 5HT3aR neurons, which belong to the reelin^+^, VIP^–^ IN population and are characterized by the expression of NDNF (Cadwell et al., 2016; Tasic et al., 2016; Abs et al., 2018; Poorthuis et al., 2018; Cohen-Kashi Malina et al., 2021). NDNF is also present in some non-NGC cells which share the same morphology as NGCs but show a reduced connectivity to L2 principal cells (Schuman et al., 2019). Therefore, in our study we were able to more specifically target the NGC subpopulation of 5HT3aR neurons than in most other functional studies of L1 interneurons. However, it should be noted that the use of viral vectors to label neurons may result in biased labeling of cellular subtypes within both 5HT3aR and NGC populations due to variations in Cre and GCaMP6 expression levels as well as differences in viral tropism between cells.

The NGC population displayed a different response than the general 5HT3aR population. Whereas 5HT3aR cells consisted of an equal fraction that up-regulated and down-regulated their activity upon learning, the NGC population displayed a larger fraction of neurons with increased activity. This suggests that conditioned tones recruit NGCs to produce a net increase in inhibitory activity on the cortical microcircuit upon presentation of a conditioned cue. The role of this presumed net increase in inhibition of pyramidal neurons by NGC in our fear learning paradigm remains unclear. This could affect integration of long-range excitatory inputs through L1 (such as from other cortical areas or thalamus), but also suppresses the output of particular pyramidal neurons. One possible interpretation would entail that increased inhibition through NGC may lead to specific suppression of outputs that normally play a role in the termination of freezing, as described in a previous study (Steenland et al., 2012). It would be interesting to investigate such a relationship by scoring freezing behavior (or general fear responses) while NGCs are imaged in head-fixed mice. Along similar lines, the decrease in activity of other neurons within the 5HT3aR population may serve to specifically promote the activity of pyramidal neurons that mediate the fear memory-related information to other brain regions such as amygdala (Skelly et al., 2017; Tipps et al., 2018) and auditory cortex (Letzkus et al., 2011).

It is also well possible that the distinct modulation of different L1 inhibitory neuron subpopulations ultimately converge to a similar effect in fear-memory formation. For example, NGCs may serve direct inhibition of pyramidal cell dendrites, whereas other 5HT3aR neurons, such as single bouquet cells (SBC), may promote disinhibition (Rudy et al., 2011; Jiang et al., 2015; Tremblay et al., 2016; Hou and Capogna, 2018; Huang and Paul, 2019). Such an effect can be envisioned since various subpopulations of 5HT3aR neurons target different components of the cortical circuits (Jiang et al., 2013; Lee et al., 2015; Markram et al., 2015; Schuman et al., 2019; Ibrahim et al., 2020; Cohen-Kashi Malina et al., 2021) and thereby exert distinct effects on pyramidal cell activity. In such distinct wiring motifs, the net upregulation of NGC activity and the net down-regulation of SBC may mutually cause a general dampening of pyramidal cell activity in ACC upon fear memory recall. Alternatively, the distinct responses may lead to inhibition and disinhibition of different cellular compartments, similar to a recent study showing that the concerted activation of VIP and PV neurons causes somato-dendritic decoupling in pyramidal neurons of the dPFC during REM sleep (Aime et al., 2022).

What could be the source of the bidirectional response modulation? In sensory cortex, higher order thalamocortical projections to L1 have been found to target inhibitory neurons such as NGCs (Pardi et al., 2020; Ibrahim et al., 2021). Along the same lines, studies in the medial prefrontal cortex indicate that projections of ventromedial (VM) nucleus of the thalamus arrive in L1a where they preferentially target NDNF cells, whereas the mediodorsal (MD) thalamus projects to L1b and targets VIP neurons (Anastasiades and Carter, 2021; Anastasiades et al., 2021). It is possible that in our paradigm the positively modulated NGCs receive preferential inputs from VM, whereas the negatively modulated 5HT3aR neurons receive inputs from the MD. Since VM likely relays information related to arousal (Schiff, 2008; Honjoh et al., 2018; Petty et al., 2021), and MD is involved in working memory (Stokes and Best, 1990; Cardoso-Cruz et al., 2013; Bolkan et al., 2017; Schmitt et al., 2017), these two pathways to the ACC may be differentially regulated during fear learning.

## Data availability statement

The raw data supporting the conclusions of this article will be made available by the authors, without undue reservation.

## Ethics statement

This animal study was reviewed and approved by the Ethics Committee of the University of Geneva and the authorities of the Canton of Geneva (license numbers: GE/28/14 and GE/61/17).

## Author contributions

GF, DK-B, AD, and AH designed the experiments and defined and built experimental setups. GF and DK-B performed the experiments and analyzed the data. JP designed and performed the cluster analysis. CC generated the transgenic NGC-Flp mouse line. EH performed the immuno-labeling characterization of NGC-Flp brains. LG managed mice colonies. RC generated the Matlab script for calcium analysis. BS implemented data analysis and interpretation. AH provided equipment and technical expertise. GF, DK-B, BS, and AH wrote the manuscript. AD and AH supervised the research. All authors except AD edited the manuscript.

## Footnotes

1
http://www.informatics.jax.org/reference/

2
http://www.informatics.jax.org/allele/

3
https://www.jax.org/strain/028584

4
http://www.scanimage.org
