# Supplementary material for: Bimodal modulation of L1 interneuron activity in anterior cingulate cortex during fear conditioning
Source: Front Neural Circuits. 2023 Jun 2;17:1138358. doi: 10.3389/fncir.2023.1138358 (PMC10272719; doi:10.3389/fncir.2023.1138358)
Supplement: Supplementary file 1 [file Data_Sheet_1.PDF]

Supplementary Information

Bimodal modulation of L1 interneuron activity in anterior cingulate cortex during fear conditioning

Giuliana Fossati†, Daniel Kiss-Bodolay†, Julien Prados, Ronan Chereau, Elodie Husi, Christelle Cadilhac, Lucia Gomez, Bianca A. Silva*, Alexandre Dayer, Anthony Holtmaat*

*** Correspondence:** Bianca A. Silva: bianca.silva@in.cnr.it,
Anthony Holtmaat: Anthony.Holtmaat@unige.ch

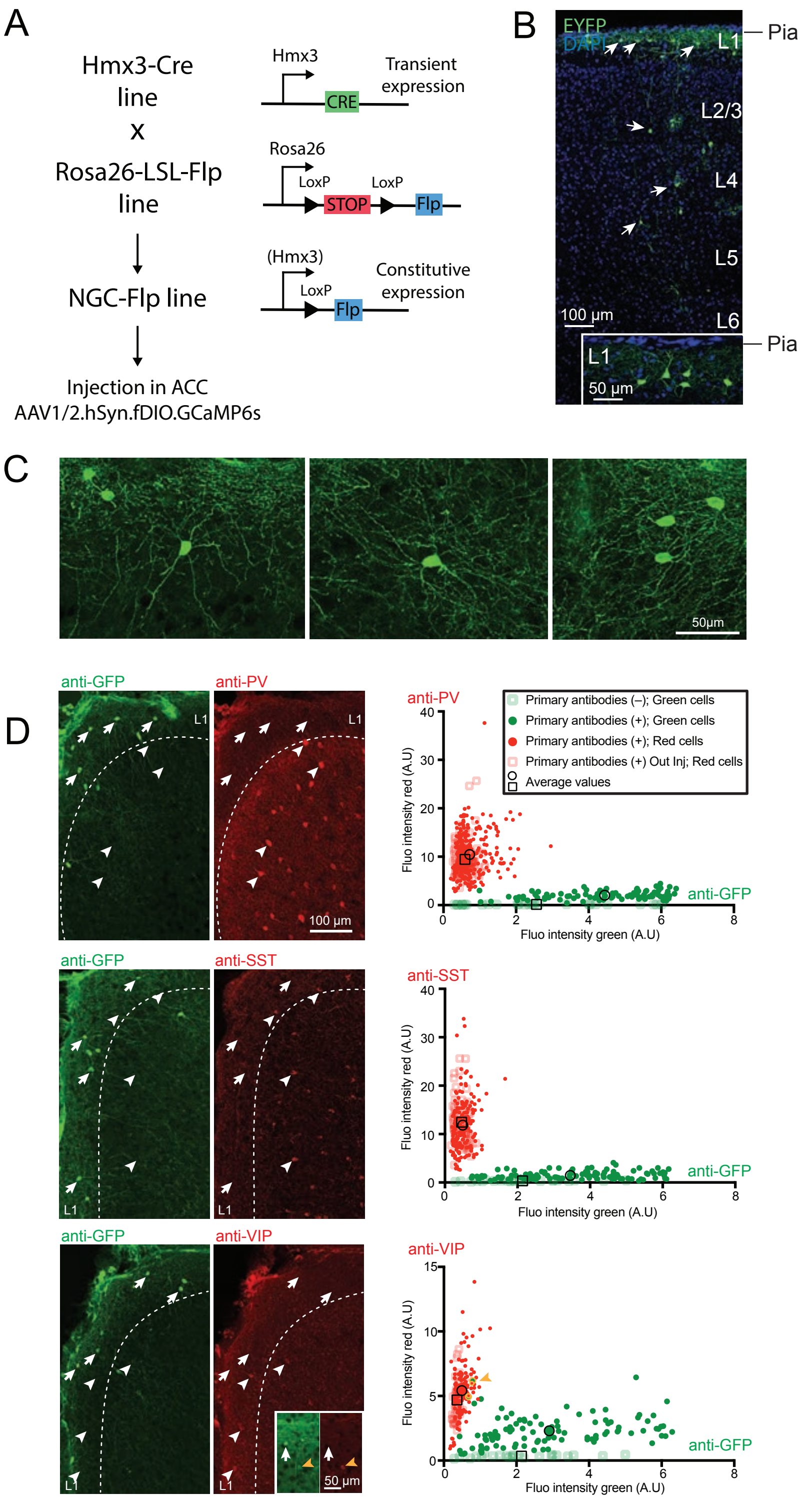

Supplementary Figure 1

<u>Figure S1:</u> NGC-Flp mouse characterization

A) NGC-Flp mice genetic intersectional strategy to create a reporter line for targeting Hmx3 derived neurons in adults. We crossed the Hmx3-Cre mouse line with a LSL-Flp mouse line that harbors a Cre-conditional Rosa-26-CAG-LSL-Flp allele. The offspring of this crossing transiently expresses Cre in NGC precursors which subsequently switches on the constitutive Rosa-26-CAG-driven expression of Flp recombinase in the mature population of NGCs.

B) The distribution of GCamMP6s-expressing cells across layers, with a relative enrichment in L1. Scale bar: 100µm. Inset: higher magnification of L1 with GCaMP6s-positive cells that have an elongated morphology, which is characteristic of NGCs. Scale bar: 50µm.

C) GCaMP6s-positive cells in L1 have highly arborized and thin axonal morphologies, which is typical for NGCs. Scale bar: 50µm.

D) Left: immuno-stained sections of the ACC from mice injected with the AAV1/2-hSyn-fDIO.GCaMP6s. The sections were co-stained with an anti-GFP antibodies (green) and antibodies against one of the inhibitory interneuron markers PV, SST or VIP (red). Arrows indicate examples of GCaMP6s-positive cells in L1 but negative for the other markers. Conversely, white arrowheads indicate cells that are positive for either anti-PV, SST or VIP but not for anti-GFP. The orange arrowhead in the inset shows an example of an anti-VIP-labeled neuron outside of L1 that is also weakly labeled for anti-GFP. Right: scatter plots showing the levels of fluorescence of individual cells that were selected either in the red or green channel. The fluorescence intensity each cell was measured in both channels. Sections were incubated with (+) or without (–) primary antibodies. A proportion of red cells were taken outside of the injected area (out of ACC), which served as controls for the background labeling in green. The plots indicate that GCaMP6s-expressing (GFP-positive) cells show negligible levels of anti-PV, SST or VIP staining. The relatively high levels of red fluorescence in green cells in the anti VIP plots is caused by fluorescent neuropil that surrounds the cells. The green fluorescent somata were therefore not selected in the red channel. Only 2 out 101 cells (orange circles) were independently selected in both channels, but they contained very low levels of green fluorescence (i.e., had negligible levels of GCaMP6s). Altogether, this indicates that GCaMP6s-expressing cells are not part of PV, SST, or VIP inhibitory classes, and strongly suggests that the mouse line allows selective targeting of NGCs. Scale bar: 100µm.

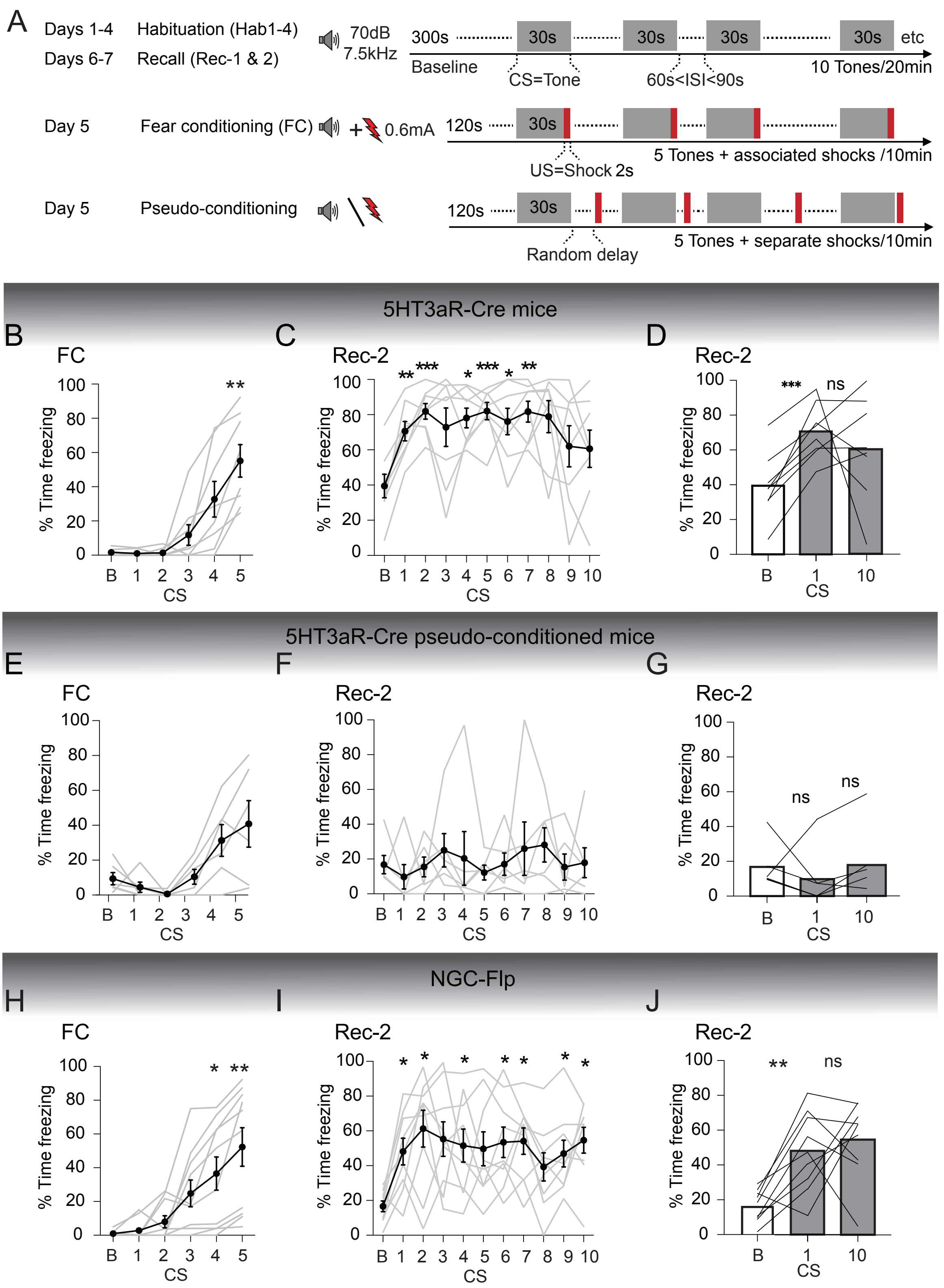

Supplementary Figure 2

<u>Figure S2:</u> Fear conditioning in microprism-implanted mice

A) Schematic representation of tone exposure during habituation, fear conditioning, and recall-sessions. Each tone is composed of 30 pips of 200ms, 1Hz (total duration: 30s, grey boxes), 70Db at 7.5kHz (10x CS). Animals are exposed to 10 tones per session after 5s of familiarization. Inter-stimulus intervals (ISIs) randomly varied between 60s-90s. For fear conditioning or pseudo-conditioning paradigms, 5x CS is delivered over 10 minutes. To achieve fear conditioning, an electrical foot shock (2s, 0.6mA) is delivered during the last of 2s of each tone (red boxes). For the pseudo-conditioning protocol, shocks and tones are uncoupled and 5 shocks are given randomly during ISIs.
B-D) Behavioural results for the 5HT3aR-Cre cohort. B) Freezing during tone presentation increased during the FC session. Freezing during the last tone was significantly higher than baseline. C) Freezing during tone presentations was significantly higher than baseline during the whole Rec-2 session in freely moving conditions. D) The percentage of time freezing during the first tone was significantly higher than baseline, while this did not decrease between the first and the last tone presentation (i.e. no within-session extinction is observed).
E-G) Behavioural results for the 5HT3aR-Cre pseudo-conditioned cohort. E) Uncoupling of CS and US leads to a delayed and not significant acquisition of the freezing state, likely due to the unpredictable nature of the electric shocks. F) CS exposure during Rec-2 did not induce increased freezing and G) freezing to CS did not change across the 10 tones presentations in Rec-2.
H-J) Behavioural results for the NGC-Flp cohort. H) Freezing during tone presentation increased during the FC session and is significantly higher than baseline in the last two tones. I) Freezing during tone presentations was significantly higher than baseline during Rec-2 session. J) The percentage of time freezing during the first tone response was significantly higher than baseline during Rec-2 and did not decrease between the first and the last tone presentation (i.e. no within-session extinction is observed).
Statistical analysis: Panels B, E, H: ANOVA $p<0.0001$. Dunnett's multiple comparisons test ***$p<0.0005$, **$p<0.005$, *$p<0.05$. Panels C, F, I: ANOVA $p<0.0001$. Dunnett's multiple comparisons test ***$p<0.0005$, **$p<0.005$, *$p<0.005$. Panels D, G, J: Paired t-test ***$p<0.0005$.

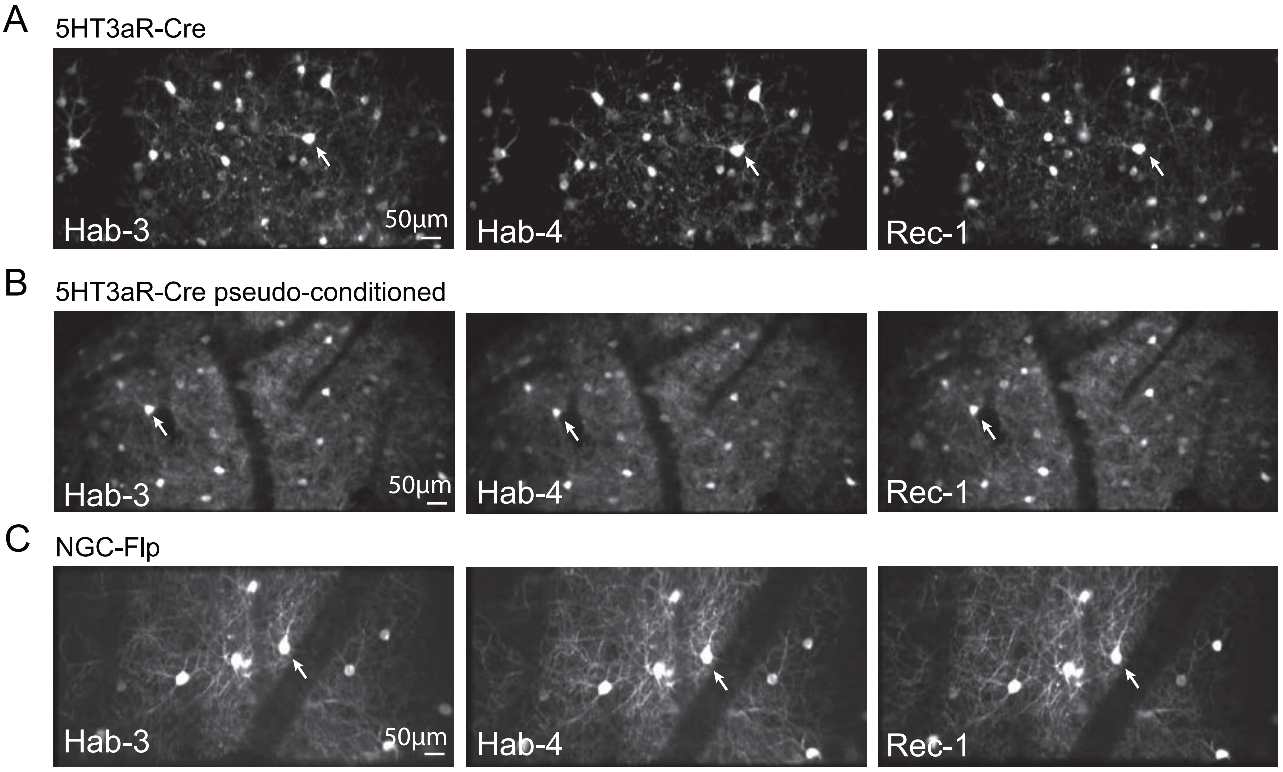

Supplementary Figure 3

<u>Figure S3:</u> Longitudinal imaging of calcium signals

Examples of longitudinally imaged neurons in 5HT3aR-Cre FC (A), 5HT3aR-Cre pseudo-conditioned (B) and NGC-Flp FC (C) mice. The same field of view and the same focal plane was imaged across days.

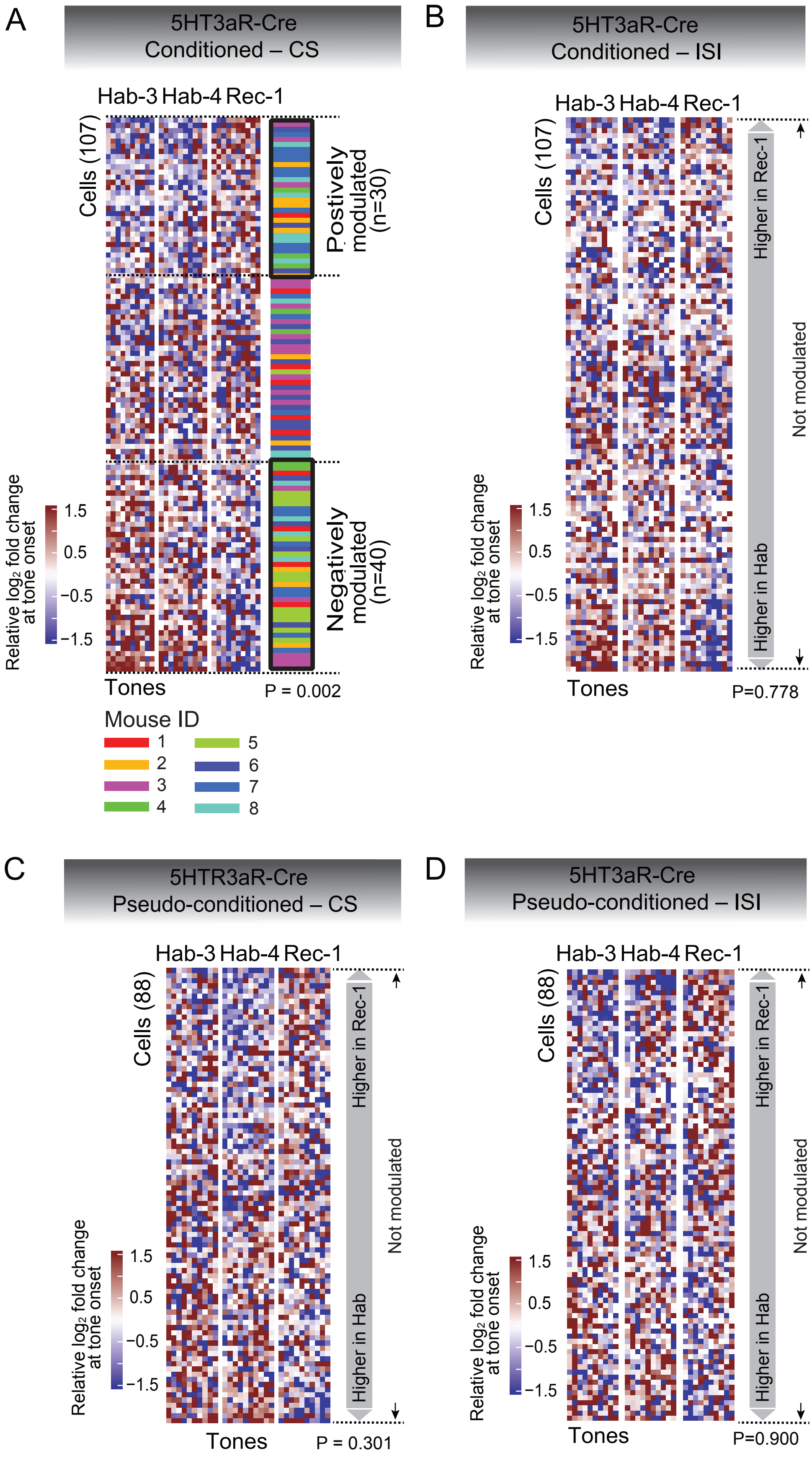

Supplementary Figure 4

<u>Figure S4:</u> The response modulation in 5HT3aR$^+$ neurons is tone- and FC-specific
A) Relative contribution of the eight experimental mice to the 107 cells used for margin analysis of the 5HT3aR-Cre dataset. B) Margin and random permutation analyses performed on random ISI intervals fails to identify modulated clusters (P=0.778), confirming that neuronal response modulation induced by fear learning is specific to tone responses. C-D) Heatmaps of pseudo-conditioned mice (tone responses, C, and ISIs responses, D). Margin and random permutation analyses revealed no clusters (C, CS, P=0.301 and D, ISIs, P=0.900).

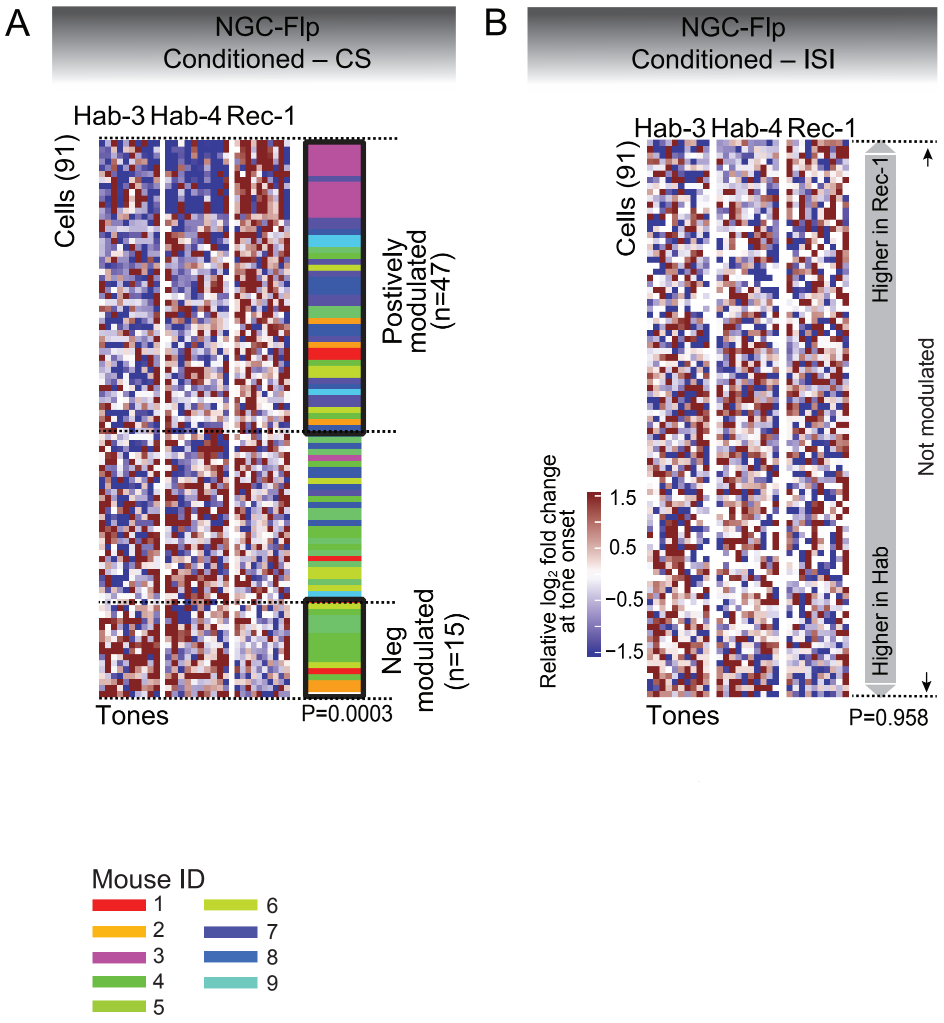

Supplementary Figure 5

<u>Figure S5</u>: The response modulation in NGCs after FC is tone-specific

A) Relative contribution of the nine experimental mice to the 91 cells used for margin analysis of the NGC-Flp dataset. Almost all mice contribute to all groups, indicating that the grouping defined by margin analysis is not due by a few outlier animals. B) Margin and random permutation analyses performed on random ISI intervals fails to identify modulated clusters (P=0.958), confirming that neuronal response modulation induced by fear learning is specific to tone responses.
